# Changes in health-related lifestyles and food insecurity and its association with quality of life during the COVID-19 lockdown in Malaysia

**DOI:** 10.1186/s12889-022-13568-0

**Published:** 2022-06-09

**Authors:** Aryati Ahmad, Mohd Razif Shahril, Nadiah Wan-Arfah, Wan Azdie Mohd Abu Bakar, Abu Bakar, Carmen Piernas, Pei Lin Lua

**Affiliations:** 1 Faculty of Health Sciences, Universiti Sultan Zainal Abidin, Kampus Gong Badak, 21300 Kuala Nerus, Terengganu, Malaysia; 2 Nuffield Department of Primary Care Health Sciences, Radcliffe Primary Care Building, University of Oxford, OX2 6GG Oxford, UK; 3 Centre for Healthy Ageing & Wellness (HCARE), Faculty of Health Sciences, Universiti Kebangsaan Malaysia, Jalan Raja Muda Abdul Aziz, 50300 Kuala Lumpur, Malaysia; 4 Department of Nutrition Sciences, Kulliyyah of Allied Health Sciences, International Islamic University Malaysia, 25200 Kuantan, Pahang, Malaysia; 5 Faculty of Pharmacy, Universiti Sultan Zainal Abidin, Kampus Besut, 22200 Besut, Terengganu, Malaysia

**Keywords:** Lockdown, Body weight, Lifestyle, Quality of life, Malaysia

## Abstract

**Background:**

The pandemic of SARS CoV2 virus has severely impacted the entire world population. The lockdown imposed during the pandemic has created enormous challenges particularly on the health, economic and social life of most individuals. This study aimed to investigate the changes in health-related lifestyle and food security during the lockdown and how they influenced the quality of life (QoL) of Malaysian adults.

**Methods:**

An online survey using a structured questionnaire consisting of sociodemographic, body weight, diet quality, physical activity, sleep quality, food insecurity, and QoL was conducted among adult respondents across Malaysia. Multivariate linear regression analyses were conducted to assess the associations between the changes in each component and QoL based on the total score before and during the lockdown.

**Results:**

A total of 759 valid responses were included in the final analysis (75% female and 24.5% male). There was a significant improvement in diet quality during the lockdown while sleep quality and food insecurity worsened significantly. As for physical activity, metabolic equivalents (METs) in moderate activity increased significantly, whilst there was a significant decrease in the METs in walking and total minutes spent on physical activity during the lockdown. Overall, independent of age, gender, ethnicity, and religion, there were significant associations between QoL during lockdown and changes in BMI, METs of moderate activity, sleep quality, and food insecurity.

**Conclusions:**

The unprecedented COVID-19 outbreak and the lockdown measure during the pandemic have caused significant negative changes in health-related lifestyles and affected the QoL of Malaysian adults. Despite the new norms and rules to prevent disease transmission, efforts to maintain a healthy lifestyle and food security among the population must be rolled out to improve the QoL and prevent further adverse mental and physical health outcomes.

## Background

The SARS-CoV-2 or coronavirus 2019 diseases (COVID-19), which was first detected in late 2019 in Wuhan, China, had caused tremendous social and economic disruptions around the globe. It has affected over 218 million of the world population with more than 4.5 million deaths worldwide [1]. This unprecedented outbreak has triggered global health concerns and most governments in many countries executed all efforts to battle the disease. Most countries including Malaysia enforced a nationwide lockdown to contain the virus from spreading, isolate the cause and flatten the chain of transmission during the peak of this pandemic in 2020. The approach to the national lockdown included a general prohibition of movements and mass gatherings across the country, barring of people from traveling abroad, strict health check-up and quarantine for citizens who return from overseas, restrictions on the entry of visitors into the country, and closure of all government and private premises except for the essential services. This implementation of lockdown has imposed impactful sequels on the physical, mental, psychological and social interactions among all populations across the world [2].

During the pandemic and lockdown, most people were confined to their homes and unable to go to work or conduct their habitual daily activities including exercising which may have associated implications on their health and wellbeing [3]. These restrictions and lifestyle changes have created stressful life events for the majority of the population and have affected the whole system and society [2, 4]. In addition to the unpleasant experience due to the loss of freedom, uncertainty over health status, and boredom which can, on occasion, create dramatic effects, it has also disrupted the chronobiological rhythms which affect the daily life routines of people including the sleep pattern leading to reduced quality of life (QoL) [3, 5]. The longer lockdown has also led to a considerable amount of stress due to inadequate supplies especially foods, financial loss, and stigma which may seriously affect the QoL [4, 6–8].

The impact of lockdown on lifestyle which may have implicated the health status of the population was worrisome in most of developed countries [9], and yet, the evidence remains scarce particularly in the developing countries. It is important to understand not only how lockdown has changed the habitual dietary intake, physical activity, food security and sleeping pattern, but also how these unwanted disruptions may have affected QoL in the population. This study aimed to determine the changes in health-related lifestyles, namely, body weight status, dietary habit, sleep quality, physical activity level, food insecurity before and during the lockdown among Malaysian adults, and the associations between these changes and QoL. This information is crucial to serving as a basis for the development of suitable guidelines and interventions both during and post-crisis in Malaysia.

## Methods

### Study design and participants

This cross-sectional survey study was conducted from May to September 2020 during the first lockdown in Malaysia. This study applied convenient sampling with Malaysian adults who have access to the internet and are able to answer questionnaires was set as the source population. Invitation to participate the study was disseminated through various communication platforms including social medias, WhatsApp, and generic email list. Malaysians aged 18 to 60 years old were included in the analysis. Adults who had been diagnosed with COVID-19 were excluded in the study.

### Data collection

A web-based online survey form was used as the main data collection method. The survey link was distributed through various social media platforms. Email invitations to participate were also conducted within the public and private organizations. Participants’ consent was obtained in the same link before the survey started. Inclusion and exclusion criteria were stated in the survey form prior to the consent.

### Questionnaires

Information regarding sociodemographic factors, socioeconomic status, health history, and self-reported weight and height were collected through the online survey platform. The online questionnaire also contained questions on four main health-related lifestyles including diet quality, sleep quality, physical activity, and food insecurity before and during the lockdown, and the overall quality of life (QoL). All variables for both timepoints were collected in the same survey during lockdown. The dietary habit was assessed using a 10-item Malaysian Healthy Diet Adherence Score which was developed based on Malaysian Dietary Guidelines [10]. Respondents recalled the serving size of each food group they consumed on a normal day and scores were calculated based on their adherence to dietary guidelines. The maximum score is 10 and higher scores indicate higher adherence to a healthy diet. Physical activity level was measured using the short Malay version of the International Physical Activity Questionnaire (IPAQ) [11]. The level of physical activity and intensity were calculated in metabolic equivalent task minutes per week (MET- minutes/week) according to the IPAQ scoring protocol. Sleep quality was measured using the Sleep Quality Questionnaire which was based on the eight-item Sleep Condition Indicator (SCI) (concerns about getting to sleep, remaining asleep, sleep quality, daytime personal functioning, daytime performance, duration of sleep problem, nights per week having a sleep problem and extent troubled by poor sleep) [12]. Meanwhile, food insecurity status was assessed using the Food Insecurity Experience Scale (FIES) questionnaire [13]. FIES is a self-reported questionnaire consisting of eight items that assess individual or household experience and behavior towards food accessibility. Participants were required to answer yes or no to all 8 questions, provided with a raw score of 0 for negative response and 1 for an affirmative response. The total FIES score is the sum of scores from all 8 questions and it is then further classified into food secure (0), mild food insecurity (1–3), moderate food insecurity (4–6), and severe food insecurity (7–8). And finally, overall QoL was measured using the 26-item version of WHOQOL-BREF [14]. In this brief version, four important domains were explored, namely physical health (7 items), psychological (6 items), social relationships (3 items), environment (8 items). For these domains, higher scores meant better QoL.

### Statistical analysis

Data were analyzed using IBM SPSS Statistics for Windows Version 26.0 software (IBM Corporation, Armonk, NY, USA). Descriptive statistics were expressed as means and standard deviation (SD) for numerical variables; frequency and percentage (%) were used for categorical variables. Data normality assumption was checked prior to analyses. Changes in body mass index (BMI), dietary habits, physical activity level, sleeping quality, and food security were calculated and analyzed using Paired t-test. The association between changes in dietary habits, physical activity level, sleeping quality, and food security and QoL were analyzed using multiple linear regression. A 5% level of statistical significance was used.

## Results

A total of 759 respondents completed a valid survey and were analyzed in this study; with 75.5% female and 24.5% male respondents. More than 50% of respondents were from the 18–30 years age group whereas equal distributions were from the age group of 31–40, 41–50, and 51–60 years old. Details of sociodemographic distributions of participants can be seen in Table 1.

**Table 1:** Participants’ sociodemographic distributions (n = 759)

	n (%)
Age Group	
18–30	391 (51.5)
31–40	142 (18.7)
41–50	109 (14.4)
51–60	117 (15.4)
Gender	
Male	186 (24.5)
Female	573 (75.5)
Marital status	
Single	414 (54.5)
Married	323 (42.6)
Divorced	22 (2.9)
Housing situation	
With partners/family	681 (89.7)
Alone	44 (5.8)
With friend	33 (4.3)
Other	1 (0.1)
Ethnicity	
Malay	449 (59.2)
Chinese	240 (31.6)
Indian	55 (7.2)
Sabah native	7 (0.9)
Sarawak native	8 (1.1)
Religion	
Islamic	456 (60.1)
Buddha	195 (25.7)
Hindu	53 (7.0)
Christian/Catholic/Taoism (to combine w Buddha)	53 (7.0)
No religion	2 (0.3)
Highest education level	
Never attend school	3 (0.4)
PMR/SRP	3 (0.4)
SPM or equivalent	53 (7.0)
STPM/Matriculation/Diploma	170 (22.4)
Degree or equivalent	427 (56.3)
Master or higher level of education	103 (13.6)
Occupation	
Professional	297 (39.1)
Support staff	106 (13.9)
Self-employed	37 (4.9)
Housewife	6 (0.8)
Pension	27 (3.6)
University/College students	277 (36.4)
Stop working/Fired	9 (1.2)
Household income	
< RM 4361	291 (38.3)
RM 4361 – RM 9619	314 (41.4)
> RM 9619	154 (20.3)
Body mass index, mean (SD)	24.19 (5.02)

Data are frequency (percentage) or mean (SD), *SD *standard deviation

All components of health-related lifestyles and food security were analyzed based on the total score before and during lockdown (Table 2). The total score of diet quality increased significantly by 0.64 (95%CI: 0.503, 0.77; *P* < 0.001) indicating diet quality was better in quality (i.e. better adherence to the Malaysian Dietary Guideline which is adequate energy and protein intake with lower salt, fat and sugar and high in fibre) during lockdown as compared to before lockdown. In terms of physical activity, METs in walking and total minutes of physical activity decreased significantly by 595.53 (95%CI: -721.44, -469.61) and 36.12 (95%CI: -46.83, -25.41) (*P* < 0.001) during the lockdown, respectively. In contrast, METs in moderate activity increased significantly during lockdown by 78.67 (95%CI: 34.30, 123.03; *P* = 0.001). Total score for sleep quality reduced by 1.94 (95%CI: -2.28, -1.59; *P* < 0.001) indicating poorer sleep quality during lockdown compared to before lockdown. Finally, the total score for food insecurity increased by 0.20 (95%CI: 0.12, 0.29; *P* < 0.001) suggesting that the respondents faced more difficulty accessing food during lockdown compared to before lockdown. The total score of QoL during lockdown was 61.38 (SD: 14.45) (Table 3). Domain 1 (physical health) had the lowest score i.e. 50.53 (SD: 12.67) whilst Domain 4 (environment) had the highest score of 69.00 (SD: 17.30).

**Table 2:** Comparison of the total score before and during MCO

Variables	Before Mean (SD)	During Mean (SD)	Mean difference	95% CI	*P*-value
Body mass index	24.19 (5.02)	24.19 (4.91)	0.003	-0.099, 0.105	0.948
Total diet quality	6.0 (2.02)	6.63 (1.82)	0.638	0.503, 0.772	< 0.001
METs in vigorous activity	571.78 (2032.92)	437.8 (909.44)	-133.987	-282.532, 14.558	0.077
METs in moderate activity	363.12 (579.68)	441.79 (789.23)	78.667	34.304, 123.029	0.001
METs in walking	1151.3 (2144.31)	555.77 (1346.73)	-595.526	-721.443, -469.610	< 0.001
Total minutes in physical activity	133.67 (171.66)	97.55 (107.34)	-36.121	-46.830, -25.412	< 0.001
Total scores for sleep quality	28.59 (5.53)	26.65 (6.47)	-1.937	-2.282, -1.591	< 0.001
Total scores for food insecurity	0.49 (1.32)	0.69 (1.45)	0.203	0.119, 0.287	< 0.001

*MCO* movement control order, *CI *confidence interval, *METs *metabolic equivalent of task, *SD *standard deviation

**Table 3:** Total score of quality of life (QOL) during lockdown

Domain	Mean (SD)
Domain 1 (Physical Health)	50.53 (12.67)
Domain 2 (Psychological Health)	58.14 (14.68)
Domain 3 (Social Relationship)	67.85 (22.37)
Domain 4 (Environment)	69.00 (17.30)
Total QoL score	61.38 (14.45)

*SD* standard deviation, *QoL *quality of life

Unadjusted analyses were conducted to determine the associations between sociodemographic, changes in health-related lifestyles, and food insecurity between before and during lockdown with QoL (Table 4). There were significant associations between changes in BMI, METs in vigorous activity, METs in moderate activity, the total score of sleep quality, and the total score of food insecurity and QoL (*P* < 0.025). Multivariable adjusted analyses showed consistent results independent of age, gender, ethnic and religion.

**Table 4:** Association between changes in health-related lifestyle and food insecurity with total quality of life

Variables	Crude regression coefficient	95% CI	*P*-value	Adjusted regression coefficient	95% CI	*P*-value
Changes in BMI	-1.554	-2.274, -0.834	< 0.001	-1.176	-1.873, -0.478	0.001
Changes in diet quality	0.083	-0.478, 0.644	0.772			
Changes in METs spent in vigorous activity	0.000	0.000, 0.001	0.225			
Changes in METs spent in moderate activity	0.003	0.001, 0.004	0.003	0.002	0.001, 0.004	0.006
Changes in METs spent in walking	0.000	0.000, 0.001	0.394			
Changes in total physical activity	0.004	-0.003, 0.011	0.322			
Changes in total scores for sleep quality	0.276	0.052, 0.499	0.016	0.406	0.193, 0.620	< 0.001
Changes in total scores to assess food insecurity	-1.117	-2.003, -0.230	0.014	-1.024	-1.870, -0.178	0.018

*CI* confidence interval. Forward multiple linear regression method was applied. Adjusted for SD - age, gender, ethnicity, religion. Model assumptions were fulfilled. R^2^ = 0.107; adjusted R^2^ = 0.099. Multicollinearity and interactions amongst independent variables were unlikely

## Discussion

The COVID-19 pandemic has unquestionably caused many life-changing events. While the impact of the virus on world infection, morbidity and mortality rates are immense, its repercussions on overall human well-being are remarkable. The safety measures imposed during pandemic, especially lockdown, have significantly deranged habitual activities and lifestyles causing adverse consequences on overall health. Although previous studies have shown the relationship between post-pandemic distress, anxiety, stress, and depression during the lockdown and mental health and decreased quality of life among global populations [5, 15, 16], the data from the Malaysian population is still lacking. This study found significant changes in total diet quality, physical activity level, sleep quality, and food insecurity level among the population during the lockdown, and some of these changes were significantly associated with QoL independent of sociodemographic factors.

 The total score of diet adherence towards dietary guidelines increased significantly indicating overall diet quality was better (i.e. adequate energy and protein intake with lower salt, fat, and sugar and high in fibre) during lockdown as compared to before lockdown. Bennett et al. [9] found there are many studies worldwide that have shown to demonstrate changes in dietary habits during lockdowns such as an increase in fresh produce, home cooking, and reduction in comfort foods, and alcohol intake. With the sudden move of local authorities to restrict movement and social interaction, accessing supermarkets to buy fresh produce and consumption of food associated with social occasions have been impacted [17]. Although there might be social and festive eating occasions within the home that are associated with consumption of low-nutritional-quality foods, this was limited as urban Malaysian families live away from their older family members who are the main driver in food preparation during social and festive seasons [18]. With the abundance of time staying at home during the pandemic, more time could be spent preparing home-cooked meals. Preparing home-cooked meals has been shown to increase diet quality as individuals become aware of the quality of ingredients and are able to plan their menu [19]. This disruption due to COVID-19 might be used as a turning point to change their dietary habits to a healthier version [20, 21]. Besides, with the increase of available information on diet and foods through the internet, many have equipped themselves with knowledge of healthy eating practices focusing on foods rich in the healthy microbiome to boost their immune system, antioxidants which are rich in fruits and vegetables as well as higher preference to eat within safe premises especially at home [22]. However, the effect of lockdown also impacted negatively on dietary practices and was associated with other unfavorable lifestyle outcomes including an increase in body weight, mental health issues, and limited physical activity [9].

In terms of physical activity, Malaysian population has shown an increase in physical activity level by nearly 19% since 2006 to 2019 [23–25]. However, METs in walking and total minutes of physical activity decreased significantly during the lockdown whilst METs in moderate activity increased significantly. Inconsistent with findings among adults in China by Wang et al. [26] where most participants did not perform moderate (jogging and dancing) and vigorous physical activity (rope jumping and weight training). This is possibly because people are not used to home workouts, have limited space, and lack motivation due to the closure of gyms [27]. Despite this, sedentary behavior has increased to 60% and people tend to eat more frequently at home because they are unable to dine in restaurants [26]. Besides, a previous study has shown that home confinement and reduced physical activity during pandemic has increased sugary food intake and levels of food cravings and snack consumptions [28]. However, although there was a decrease in the physical activity level during the lockdown in this study, the reason behind the reduction was not further investigated. Malaysians are encouraged to work from home and restrict outdoor activities since the imposed stay-at-home order which was one of the obvious reasons behind the decrease in physical activity. Our findings were also in line with a study conducted among Brazilians during the pre-pandemic period where less than 5% of participants were inactive and the remaining equally moderately and highly active. However, in the pandemic period, 84% of the sample population was considered inactive [29]. Based on a study by Tison et al. [30], there was a 5.5% average decrease in daily step count (287 steps) worldwide following a step counter smartphone application within 10 days after the March 11th pandemic started. Furthermore, the inactivity increased to 27.3% (1432 steps) within 30 days. Meanwhile, in China, the average steps per day and the average moderate or vigorous-intensity exercise also declined significantly for both males and females during the semi-lockdown [31]. The restriction of movement due to the pandemic had changed work and transport-related physical activities for a large number of the working population and affected leisure activities by the closure of sports and fitness centers.

The COVID-19 lockdown was also associated with significant changes in other daily activities including sleep schedule. The psychological distress, anxiety, and depression during pandemic and lockdown have disrupted sleeping patterns leading to poor quantity and quality of night-time sleep [32]. This study found that sleep quality has dropped significantly during the lockdown as compared to the pre-lockdown among the Malaysian population. In India, COVID-19 lockdown has shifted the bed- and waking timing, reduced the night-time sleep and increased daytime napping, and caused sleep quality deterioration [3]. Similar findings were also found among populations in European countries including Spain, Italy, and Belgium [33]. In addition to physical health, impaired sleep quality can lead to substantial negative psychological and physiological consequences including increased stress responsivity, somatic pain, emotional distress and mood disorders, and cognitive, memory, and performance deficits which ultimately will lead to reduced QoL [34].

The longer lockdown has notably impacted food security around the globe due to unemployment, and disruption in food supply and accessibility. The World Food Summit defined that “food security exists when all people, at all times, have physical and economic access to sufficient, safe and nutritious food that meets their dietary needs and food preferences for an active and healthy life” [35]. The current study shows a significant increment in food insecurity score during the lockdown period compared to before the lockdown period indicating that people were more food insecure during the lockdown period. Our findings are in line with other surveys from both developed and developing countries [36–38]. Undeniably, the COVID-19 pandemic among others had caused economic and psychological vulnerability which had influenced the food security status [36, 39]. Many have turned to food banks or free food distribution to obtain their basic food needs, other than relying on financial aid from the government [40]. This situation if left unattended could worsen and may lead towards more severe health consequences as food insecurity is associated with anxiety and depression as well as psychological distress during the current pandemic [41–43].

Across all respondents, this study found that QoL was, in general, at a moderate level during the COVID-19 lockdown period, hence satisfactory. A similar outcome was reported by a study among over 2,000 adults in China who had been isolated at home for an average of 77 days [26]. Approximately 65% of the respondents indicated being satisfied with their QoL. However, the physical domain was comparatively the worse whilst the environmental dimension was the best. This was unsurprising in view of the significant contribution of physical activity (and intensity) to QoL as measured in METs which was proven to be independent of several important socio-demographic variables in our sample. A recent China study also showed the negative impact of the COVID-19 pandemic towards participation in physical activity, which was in turn significantly associated with QoL [6]. The increase in sedentary time and reduction in outdoor physical movement as a consequence of home confinement which has been reported [44–46] were the main attributes of such findings. Supporting this, Wang and co-researchers [47] specifically pointed out that 50% of their stay-home Chinese respondents had diminished time for physical activity while over 60% experienced increased sedentary behavior. It has been contended that people’s engagement with physical activity could stem from one’s motivation and self-efficacy [48]. Moreover, the less active Canadians in their study reported significantly reduced benefit, enjoyment, confidence, support, and opportunities to continue an active lifestyle in the COVID-19 restriction period. Given the protective benefits of physical activity on health and well-being, the government should at any feasible point permit the continuity of this activity at all levels to ensure sustained personal motivation and self-efficacy.

On the other hand, the more favorable outcome on environmental issues was a likely expectation as people generally feel safer at home amid an infectious pandemic such as COVID-19. In addition, the majority were also staying with family members, and spending such interactive time together (cooking, watching tv, chatting, etc.) was naturally a positive surrounding, at least temporarily. The social relationship was also reported to be relatively better when compared to the physical and psychological dimensions, as more time was spent to rest and reconnect with family and friends (via social media, if not physically). Enhanced shared feelings and family care was encouragingly documented [5]. The same picture regarding environmental and social aspects was also depicted by another study among a bigger sample of respondents [26]. Nonetheless, negative changes in weight [49, 50] physical activity level [46], sleep quality [3, 51, 52], and food insecurity [41, 53] which occurred during lockdown may have introduced psychological distress that lead to significant influence on general QoL. Although the non-significant changes in body weight during lockdown may not reflect the true changes, however every unit change significantly impacted the QoL after controlling for other factors.

This study was the first study to report the impact of lockdown on health-related lifestyles and how it affects QoL among Malaysian adults. The findings of this study nationally represent the Malaysian adults from the major ethnicities, however, the cross-sectional study design cannot be used to infer causality. While the sample sizes may be smaller than we anticipated, our survey was also limited by its online nature and may be biased towards the more educated and technology-savvy population. There was a potential of misreporting errors from the self-reported data especially when both before and during variables were collected in the same survey during lockdown. The number of variables collected were also limited which may had introduced the effect of residual confounding in the associations. The fact that this study used convenient sampling, the percentage of responses was unable to be reported. Nevertheless, the present findings highlighted the importance of lifestyle on QoL and how the lockdown measure during the pandemic had caused significant changes. The government and health authorities should consider appropriate interventions to prevent further disruptions in life and increase wellbeing to increase the QoL of the population, especially during lockdown and post-pandemic. As the world is facing transition to endemic state, creating safe environment while rebuilding the economic and health of the nations is pivotal.

## Conclusions

Lockdown measure had caused significant changes in lifestyles among Malaysian adults which had influenced their QoL. Increased BMI and food insecurity, and poor sleep quality were associated with reduced QoL, whilst increased in moderate physical activity during lockdown increased QoL among Malaysian adults. These findings have confirmed that lockdown caused a significant impact on human wellbeing and therefore suitable interventions particularly psychological interventions and physical accessibility to health and lifestyle resources are recommended to prevent further impairment of QoL.

## Data Availability

The datasets used and analyzed during the current study are not openly available due to the sensitivity of personal data and are available from the corresponding author on reasonable request.

## Abbreviations

QoL: Quality of life
METs: Metabolic equivalents
BMI: body mass index
IPAQ: International Physical Activity Questionnaires
SCI: Sleep Condition Indicator
FIES: Food Insecurity Experience Scale
CI: confidence interval
SD: standard deviation
